# Inactivation of *O^6^-methylguanine-DNA methyltransferase* by promoter CpG island hypermethylation in gastric cancers

**DOI:** 10.1038/sj.bjc.6600372

**Published:** 2002-06-17

**Authors:** S I Bae, H S Lee, S H Kim, W H Kim

**Affiliations:** Department of Pathology, Seoul National University College of Medicine, 28 Yongon-dong, Seoul 110-799, Korea; Cancer Research Institute, Seoul National University College of Medicine, 28 Yongon-dong, Seoul 110-799, Korea

**Keywords:** *O^6^-methylguanine-DNA methyltransferase*, immunohistochemistry, survival analysis, stomach neoplasms, DNA methylation

## Abstract

Promoter hypermethylation of CpG islands in tumour suppressor genes can lead to transcriptional inactivation. To investigate the association between methylation and expression at *O^6^-methylguanine-DNA methyltransferase*, we performed methylation-specific PCR and immunohistochemistry in 149 gastric carcinomas. Promoter methylation was found in 14.1% of tumours and loss of expression was detected in 11.4% of tumours. To examine correlation between the *O^6^-methylguanine-DNA methyltransferase* expression and the clinical data, we investigated *O^6^-methylguanine-DNA methyltransferase* expression in 315 consecutive gastric carcinomas. A similar frequency of loss of *O^6^-methylguanine-DNA methyltransferase* expression was confirmed in these cases. The loss of *O^6^-methylguanine-DNA methyltransferase* expression was significantly associated with pTNM stage (*P*=0.037), tumour invasion (*P*=0.02), microsatellite instability (*P*=0.041) and overall survival (*P*=0.01). Among 11 gastric cancer cell lines, SNU-620 showed the loss of *O^6^-methylguanine-DNA methyltransferase* expression as well as promoter methylation. After treatment with 5-aza-2-deoxycytidine, a demethylating agent, SNU-620 re-expressed *O^6^-methylguanine-DNA methyltransferase* mRNA. In summary, we suggest that during gastric carcinogenesis, the loss of *O^6^-methylguanine-DNA methyltransferase* expression frequently occurs via the hypermethylation of the CpG islands of the promoter region, and that this is significantly associated with the clinicopathological characteristics.

*British Journal of Cancer* (2002) **86**, 1888–1892. doi:10.1038/sj.bjc.6600372 www.bjcancer.com

© 2002 Cancer Research UK

## 

Epigenetics is defined as an inheritable effect that influences gene activity but does not involved a change in the DNA sequence. DNA methylation is one of these epigenetic changes in human cancer and is involved in gene silencing. About 60% of human genes have CpG islands at the 5′ region of their DNA sequence. This region contains the promoter and the exons and is normally unmethylated (Toyota *et al*, 2000). Aberrant methylation of CpG islands in tumour suppressor genes can lead to transcriptional inactivation. Promoter hypermethylation of CpG islands of tumour suppressor genes (*p16*, *p14*, and *APC*), DNA repair genes (*hMLH1* and *MGMT*) and genes related to metastasis and invasion (*E-cadherin*, *TIMP-3*, and *DAPK*) has been shown in various cancers and is associated with the loss of expression (Yoshiura *et al*, 1995; Herfarth *et al*, 1999; Eads *et al*, 2001; Esteller *et al*, 2001a; Rosas *et al*, 2001 Zöchbauer-Müller *et al*, 2001).

*O^6^-methylguanine-DNA methyltransferase* (*MGMT*) is a DNA repair gene, which removes methyl groups as well as larger adducts at O^6^ position of guanine. *MGMT* transfers the alkyl group from O^6^-guanine in DNA to an active cysteine within its own sequence in a reaction that inactivates one *MGMT* molecule for each lesion repaired (Pegg, 1990; Esteller *et al*, 1999). Methylating agents such as N-methyl-N-nitrosourea (MNU) and N-methyl-N′-nitro-N-nitrosoguanidine (MNNG) react with guanine in DNA to form O^6^-methylaguanine. The alkylation of DNA at the O^6^ position of guanine is associated with formation of DNA mutation in cancers, such as the transition of guanine-cytosine to adenine-thymine pairs in the *K-ras* mutation or in *p53*, which occurs because alkylated guanine is recognised as adenine during DNA replication (Esteller *et al*, 2000a, 2001b; Whitehall *et al*, 2001).

Methylation of the *MGMT* gene has been reported in various carcinomas. In gliomas and colorectal cancers, methylation was shown in 38% of the tumour, whereas in non-small cell lung carcinomas, lymphomas, and head and neck carcinomas, methylation was demonstrated in 23–28% of tumours (Esteller *et al*, 1999; Rosas *et al*, 2001). Promoter hypermethylation of *MGMT* in colorectal carcinomas results in transcriptional inactivation of *MGMT* gene (Esteller *et al*, 2000a).

We examined the loss of expression and the promoter methylation of *MGMT* in 149 gastric carcinomas and 11 gastric cancer cell lines and investigated an association with loss of expression and clinicopathological characteristics in consecutive gastric carcinomas.

## MATERIALS AND METHODS

### Primary gastric cancer tissue samples

Initially, 149 stomach carcinomas and matched normal tissues were obtained from surgical resection specimens at Seoul National University Hospital from 1998 to 1999. All samples were fixed using absolute methanol, processed in chloroform and DNA was extracted by the phenol-chloroform methods. Formalin-fixed, paraffin embedded samples were arranged into three tissue array blocks. In addition to the 149 stomach carcinoma specimens, 315 consecutive cases of formalin-fixed, paraffin embedded stomach specimens were arranged into six tissue array blocks (Lee *et al*, 2001). None of the patients received preoperative chemo- or radiotherapy. These array blocks were cut into 4 um sections, and deparaffinized and dehydrated. Immunohistochemistry was carried out using a Vector ABC kit (Vector Laboratories, Inc., Burlingame, CA, USA) and mouse anti-MGMT monoclonal antibody (Chemicon, Temecula, CA, USA) at a dilution of 1 : 50.

### Gastric cancer cell lines

Eleven gastric cancer cell lines, SNU-1, -5, -16, -216, -484, -520, -601, -620, -638, -668, and -719, were obtained from the Korean Cell Line Bank (Seoul, Korea). They were cultured in RPMI-1640 (Life Technologies, Rockville, MD, USA) supplemented with 10% heat-inactivated FBS (Hyclone, Logan, UT, USA).

For Western blot analysis, cell lines were harvested with PBSTDS containing 1% Trition X-100, 0.5% sodium deoxycholate, and 0.1% SDS. Equal amounts of protein, as determined by a bicinchoninic acid (BCA) assay (Pierce, Rockford, IL, USA), were dissolved in sample buffer, separated by electrophoresis on 12% SDS-polyacrylamide gel and transferred to a polyvinylidene difluoride membrane (Millipore Corp., Bedford, MA, USA). The membrane was blocked with 3% non-fat dry milk in tris buffer-saline with 0.1% Tween 20 for 1 h at room temperature and incubated with the mouse anti-MGMT monoclonal antibody (1:500, Chemicon) at 4°C overnight. The membrane was then washed in the same buffer, incubated in horseradish peroxidase conjugated anti-mouse antibody (Amersham Pharmacia, Buckinghamshire, UK) for 30 min at room temperature, and then immersed in an enhanced chemiluminescence (ECL) Western blotting detection system (Amersham Pharmacia).

For reverse transription-PCR, total RNA of the gastric cancer cell lines was extracted using Trizol (LifeTechnologies) and 0.5 ug of the total RNA was used to generate cDNA. This cDNA was amplified with primers, 5′-GTG GGA GGA GCA ATG AGA GG-3′ and 5′-TCC CGC TCC CTT GAG CCA GG-3′ (Watts *et al*, 1997). The primers for β*-actin*, the positive control, were 5′-ACA CTG TGC CCA TCT ACG AGG-3′ and 5′-AGG GGC CGG ACT CGT CAT ACT-3′. The PCR products obtained were analysed by 1.5% agarose gel electrophoresis with ethidium bromide and examined under UV light.

In order to demethylate the methyl-CpG sites of DNA, cell lines were cultured in a 100 mm culture dish (Nunc, Roskilde, Denmark) and were treated with 10 uM of 5-aza-2′-deoxycytidine (Sigma, St. Louis, MO, USA) for 10 days.

### Methylation-specific PCR

DNA modification was carried out according to the procedure described by Frommer *et al* (1992). One ug of DNA was denatured for 5 min at 94°C, 10 ul of 1 N HCl was then added, and the mixture was incubated for 10 min at 37°C. The denatured DNA obtained was modified using 3.5 M sodium bisulphite per 1 mM hydroquinone (pH 5.0) for 16 h at 50°C, and the modified DNA were then purified using a Wizard DNA clean-up system (Promega, Madison, WI, USA). Fifteen ul of 1 N HCl was added to the purified DNA, which was then precipitated with ethanol, and resuspended in 20 ul of water.

After the sodium bisulphite modification, the DNA was amplified in a volume of 10 ul with methylation specific primers (Esteller *et al*, 1999). The primers for methylated DNA were sense 5′- TTT CGA CGT TCG TAG GTT TTC GC-3′ and antisense 5′-GCA CTC TTC CGA AAA CGA AAC G-3′. The primers for unmethylated DNA were sense 5′-TTT GTG TTT TGA TGT TTG TAG GTT TTT GT-3′ and antisense 5′-AAC TCC ACA CTC TTC CAA AAA CAA AAC A-3′. The amplification conditions were as followed: 95°C for 5 min; followed by 33 cycles of 94°C for 30 s, 59°C for 30 s, and 72°C for 40 s; followed by final extension of 72°C for 10 min. PCR products were electrophoresed on 6% polyacrylamide gel, stained with ethidium bromide, and visualised under UV illumination (Herman *et al*, 1996).

### Sodium bisulphite genomic sequencing

After the sodium bisulphite modification, the DNA was amplified using hemi-nested primers. The primers were sense, 5′-TTA AGG TAT AGA GTT TTA GGC GGA AGT TGG-3′, nested sense, 5′-TTT AGC GAG GAT GTG TAG ATT GTT TTA GGT-3′, and antisense, 5′-AAA ACG AAA CGA CCC AAA CAC TCA CCA AAT-3′ (Qian and Brent, 1997). The amplification conditions were as followed: 95°C for 5 min; followed by 33 cycles of 94°C for 30 s, 62°C for 30 s, and 72°C for 40 s; followed by final extension of 72°C for 10 min. The first round PCR products were purified with High Pure PCR product purification kit (Boehringer Manheim, Mannheim, Germany). The final hemi-nested PCR products were amplified at an annealing temperature of 64°C. The amplified PCR products were purified using a PCR product pre-sequencing kit (Amersham Pharmacia) and sequencing was carried out with a BigDye Terminator Cycle Sequencing Ready Reaction kit (Perkin Elmer, Foster City, CA, USA) using an ABI Prism 377 DNA sequencer (Perkin Elmer).

### Statistics

Statistical analysis was performed using SPSS 9.0 software (SPSS, Chicago, IL, USA). Associations between the discrete variables were assessed using the two-sided Fisher's exact test or Pearson's chi square tests. Overall survival was calculated by Kaplan–Meier log-rank test. Multivariate analysis to assess the independent prognostic value of MGMT staining was performed by Cox regression. *P* value less than 0.05 was regarded as statistically significant.

## RESULTS

### Promoter methylation and loss of MGMT expression in 149 gastric carcinomas

**Figure 1:**
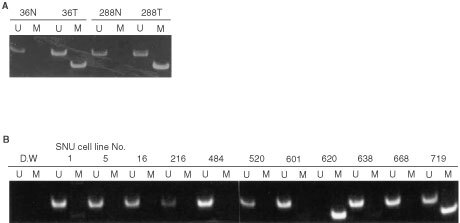
Methylation-specific PCR result of *MGMT* in primary gastric carcinomas and the gastric cancer cell lines. (**A**) In gastric carcinomas, matched normal tissues (N) showed only unmethylated bands but tumours (T) showed both unmethylated and methylated bands. (**B**) The SNU-620 cell line showed only the methylated allele but the SNU-719 cell line showed both methylated and unmethylated alleles. Methylated product was not detected in other cell lines. U, unmethylated allele; M, methylated allele.

**Figure 2:**
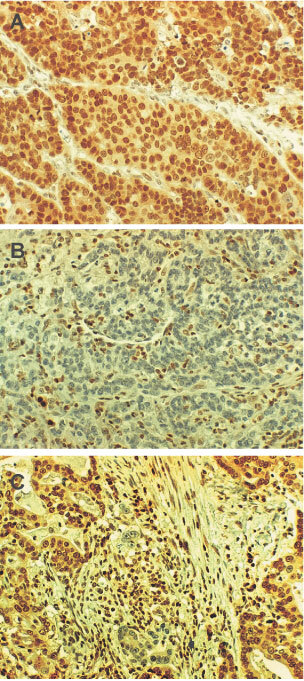
Expression of MGMT in gastric carcinomas. On immunohistochemistry, MGMT protein expressed in the nuclei of normal cells and cancer cells (**A**). In some cases, nuclear MGMT expression is lost completely (**B**) or focally (**C**).

To examine promoter methylation, we carried out methylation-specific PCR in the 149 methanol-fixed gastric carcinomas. Methylation was detected in 14.1% (21/149) of tumours. None of the matched normal tissues showed methylated bands (Figure 1A

). To investigate expression, we applied tissue array method and carried out immunohistochemistry in formalin-fixed gastric carcinomas. MGMT protein was normally expressed in the nucleus of most parenchymal and stromal cells (Figure 2A

). Seventeen cases (17/149, 11.4%) of tumours showed complete loss of MGMT expression (Figure 2B) and 13 cases of these (76.5%) were methylated in promoter region. Out of the 132 tumours with MGMT expression, eight tumours (6.1%) showed methylation, and among these eight cases, three showed loss of MGMT expression in the focal area of the tumour (Figure 2C). In chi square test, promoter hypermethylation of *MGMT* was significantly associated with a loss of expression in gastric carcinomas (Table 1

**Table 1:**
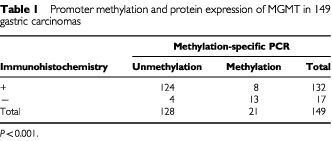
Promoter methylation and protein expression of MGMT in 149 gastric carcinomas

, *P*<0.001).

### Loss of expression and clinicopathological data in consecutive gastric carcinomas

To investigate the association between the loss of MGMT expression and the clinicopathologic characteristics, we carried out immunohistochemistry using six tissue array blocks containing 315 consecutive gastric carcinomas with the follow-up data. Loss of MGMT expression was found in 13.3% of tumours and was significantly associated with pTNM stage (*P*=0.037), and tumour invasion (*P*=0.02). We previously investigated microsatellite status using BAT-25 and BAT-26 markers in 315 gastric cancers (accepted in *Modern Pathol*). Loss of MGMT expression was significantly associated with microsatellite instability (*P*=0.041) (Table 2

**Table 2:**
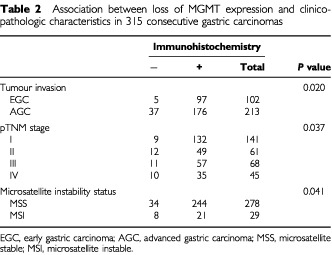
Association between loss of MGMT expression and clinicopathologic characteristics in 315 consecutive gastric carcinomas

). Furthermore, patients with the loss of MGMT expression had a poorer prognosis than those with a normal expression pattern (*P*=0.01) (Figure 3

**Figure 3:**
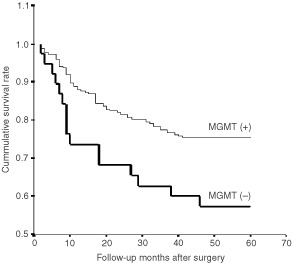
Overall survival of the gastric cancer patients according to MGMT expression. Cases with loss of MGMT expression showed poor prognosis compared to those with normal expression pattern (*P*=0.01).

). In the multivariate analysis, MGMT did not demonstrate a correlation with survival, when simultaneously assessed with age, stage, histology, and lymph node metastasis (*P*=0.16).

### Promoter methylation and loss of expression in gastric cancer cell lines

**Figure 4:**
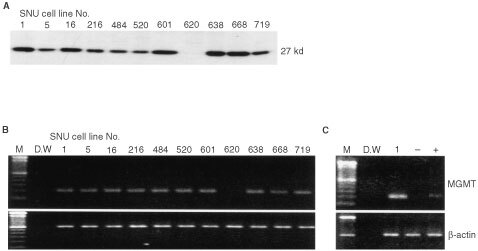
Expression of MGMT in gastric cancer cell lines. (**A**) Protein expression by Western blot analysis. All but SNU-620 cell line express MGMT protein. (**B**) mRNA expression by RT–PCR. *MGMT* mRNA was not detected in the SNU-620 cell line. β-actin was used as a control. (C) *MGMT* mRNA expression after 5-aza-2′-deoxycytidine treatment. The SNU-620 cell line expressed mRNA after 10 uM of 5-aza-2′-deoxycytidine treatment for 10 days. The SNU-1 was used as a positive control.

Promoter methylation was detected in the SNU-620 and SNU-719 cell lines. The SNU-620 cell line contained only methylated allele but SNU-719 contained both unmethylated and methylated alleles (Figure 1B). To compare MGMT expression *vs* methylation, Western blot analysis and RT–PCR were performed. The protein and the mRNA of *MGMT* were absent in the SNU-620 cell line (Figure 4A,B

). To confirm the methylation status of the CpG site, we performed bisulphite genomic sequencing of the nt −128 to −44 promoter region. In the SNU-620 cell line, which contained only the methylated allele, 9 of 14 CpG sites (−125, −122, −106, −90, −74, −70, −63, −52, and −46) were found to be completely methylated (data not shown). The significance of the methylation status upon MGMT expression *in vitro* was confirmed by adding a demethylating agent to SNU-620, which does not express *MGMT* mRNA. After 10 days of treatment with 10 uM 5-aza-2′-deoxycytidine, *MGMT* mRNA was detected by RT–PCR (Figure 3C).

## DISCUSSION

Normal tissues exhibit methylation in CpG sites in exons randomly and global hypomethylation occurs in these methylated CpG sites during carcinogenesis (Toyota *et al*, 2000). In contrast, CpG sites in the promoter regions of many tumour suppressor genes are normally unmethylated but are heavily methylated in cancer tissue (Esteller, 2000b). In the present study, promoter methylation was detected in 14.1% of gastric carcinomas and a loss of MGMT expression was found in 11.4–13.3% of tumours. Our result is lower than those of Oue *et al* (2001) or Park *et al* (2001) but is similar to those of Esteller *et al* (2001a). The study by Park *et al* (2001) showed that there was no significant difference in the protein expression between cases with *MGMT* methylated and unmethylated tumours. But in our results, as well as the study by Oue *et al* (2001), there were significant differences associated between the loss of MGMT protein expression and promoter methylation (*P*<0.001).

Among the cases with a loss of MGMT expression, 76.5% were methylated while 6.1% of tumours were methylated in cases showing MGMT expression. Furthermore in tumours showing both MGMT expression and promoter methylation, three cases showed loss of expression in the focal area of the tumour, i.e., 1 or 2 glands in intestinal type or a small area in diffuse type (Figure 2C). Because MGMT protein was expressed in more than 10% of the above cases, they were regarded positive. Due to high sensitivity of methylation-specific PCR, the methylated band may be observed even if the methylated DNA represents only a minor portion. In the 315 consecutive cases examined, 12 cases showed focal loss of MGMT expression and eight of these cases were at stage I or II. Therefore, it appears that during carcinogenesis, methylated cancer cells in a small region may expand clonally, and subsequently dominate the whole tumour tissue.

To investigate an association between MGMT expression and clinicopathological characteristics, we additionally investigated MGMT expression in consecutive cases and compared the results with clinicopathologic data. Cases with loss of MGMT expression were associated with advanced gastric cancer (*P*=0.02) and microsatellite instability (*P*=0.041) and cases with MGMT expression were associated with longer survival (*P*=0.01).

Recently, several studies concerning the relations between *MGMT* and MSI was reported (Whitehall *et al*, 2001; Laiho *et al*, 2002) and Yamamoto *et al* (2002) revealed that the sporadic colorectal cancers with MSI-H showed frequently methylation pattern in various genes and showed *MGMT* promoter methylation in 23% of the cases. In our gastric cancer cases, the loss of MGMT expression is significantly associated with microsatellite instability (*P*=0.041) as well as with hMLH1 expression (*P*=0.002, data not shown). Our results may suggest that cancers with simultaneous methylation of CpG islands, so-called CpC islands methylator phenotype or CIMP+, may demonstrate the promoter methylation of both *MGMT* and *hMLH1* genes. In addition, cells with loss of MGMT expression are left unprotected from mutagens and may predispose to additional mutations in the genes involved in the progression of tumours which may lead to advanced stage and poor survival.

In SNU-620 cell line, which contains only methylated allele, loss of MGMT protein and mRNA expression were reversed with treatment of 5-aza-2′-deoxycytidine, a demethylating agent. It may support that the promoter methylation of MGMT could silence the protein expression in gastric cancer cells.

In summary, we suggest that during gastric carcinoma progression, loss of expression at the *MGMT* gene is frequently caused by promoter hypermethylation of CpG islands and that this is significantly associated with tumour progression and prognosis.
